# Prevalence and Fall Risk of Sarcopenia Based on the 2023 Korean Working Group on Sarcopenia Criteria

**DOI:** 10.3390/medicina61061065

**Published:** 2025-06-10

**Authors:** Minjung Kim, Seongmin Choi, Dong Hwan Yun, Yunsoo Soh, Chang Won Won

**Affiliations:** 1 Department of Rehabilitation Medicine, Seoul National University Hospital, Seoul 03080, Republic of Korea; koko9638@naver.com; 2 Department of Rehabilitation Medicine, Seoul National University Bundang Hospital, Seongnam 13620, Republic of Korea; ysisminee@naver.com; 3 Department of Physical Medicine and Rehabilitation, Kyung Hee University Medical Center, College of Medicine, Kyung Hee University, Seoul 02447, Republic of Korea; dhyun@khu.ac.kr; 4 Department of Family Medicine, Kyung Hee University Medical Center, College of Medicine, Kyung Hee University, Seoul 02447, Republic of Korea

**Keywords:** sarcopenia, functional sarcopenia, fall, sex differences, Korean Working Group on Sarcopenia

## Abstract

*Background and Objectives*: Sarcopenia is a major risk factor for falls in older adults. The 2023 Korean Working Group on Sarcopenia (KWGS) introduced revised definitions, including functional sarcopenia, which considers low strength and performance despite normal muscle mass. This study investigated the prevalence of sarcopenia, severe sarcopenia, and functional sarcopenia using the KWGS criteria and their association with fall risk by sex and fall frequency. *Materials and Methods*: A cross-sectional analysis was conducted using data from 2061 community-dwelling Korean adults aged 70–84 years who participated in the Korean Frailty and Aging cohort study. Sarcopenia was classified based on muscle mass, grip strength, and four physical performance tests. Fall experiences in the past year were categorized as ≥1, ≥2, and ≥4 falls. Logistic regression analyses were performed separately according to sex to evaluate the association between sarcopenia definition and fall risk. *Results*: The prevalence of sarcopenia and severe sarcopenia was 32.9% and 10.1% in men and 21.5% and 5.0% in women, respectively. Functional sarcopenia was more prevalent in women (10.5%) than in men (5.1%). In men, sarcopenia (defined using gait speed) was associated with fall risk across all thresholds (odds ratio [OR] = 2.28 for ≥1 fall; OR = 5.64 for ≥4 falls). In women, sarcopenia (defined using gait speed) was associated with ≥1 fall (OR = 1.72), while functional sarcopenia (defined using gait speed and timed up-and-go test) was associated with frequent falls (OR = 3.79–3.87). *Conclusions*: The 2023 KWGS guidelines revealed sex-specific differences in the prevalence of sarcopenia and highlighted gait speed as a key predictor of fall risk in men, whereas functional sarcopenia was more prevalent in women. Limitations include the cross-sectional design and use of self-reported fall data, which may be subject to recall bias.

## 1. Introduction

Sarcopenia is a progressive condition that affects skeletal muscles, leading to a rapid decline in muscle mass and physical function. It is increasingly being recognized as an important health concern owing to its association with frailty, functional impairment, falls, and mortality [1]. Despite its clinical importance, universally accepted definitions, standardized diagnostic criteria, and treatment guidelines have not yet been established [2]. Since the initial conceptualization of sarcopenia, expert organizations have developed and refined the diagnostic frameworks. Earlier definitions focused on muscle mass while overlooking functional aspects [3,4]. More recently, international organizations have revised their criteria to incorporate muscle function as a key diagnostic component [5]. Of these, the European Working Group on Sarcopenia in Older People (EWGSOP) introduced its first diagnostic guidelines in 2010, although they did not include defined cutoff values [6].

In Asia, the Asian Working Group for Sarcopenia (AWGS) formulated diagnostic criteria based on the EWGSOP guidelines, introducing specific cutoff values that integrated muscle mass, strength, and physical function. In 2019, AWGS expanded the concept by introducing “possible sarcopenia”, aimed at facilitating early diagnosis, particularly in primary care settings [7]. In 2023, the Korean Working Group on Sarcopenia (KWGS) adapted and refined these criteria to streamline the diagnostic process. Their approach introduced the concept of “functional sarcopenia”, which identifies individuals with reduced muscle strength and physical function, despite having normal muscle mass. Additionally, KWGS established clearer diagnostic thresholds [8].

Studies have suggested that older adults experiencing multiple falls are at greater risks of functional deterioration, impaired mobility, and severe injuries [9,10]. Although sarcopenia has been widely linked to falls and fractures, the strength of this association varies, depending on the diagnostic criteria used. A recent meta-analysis found that sarcopenia diagnosed using the EWGSOP and IWGS criteria was strongly associated with increased fall risk, whereas this association was weaker or absent based on the Foundation for the National Institutes of Health definition [11,12]. Given the ongoing revisions in the diagnostic frameworks, numerous studies have investigated the prevalence of sarcopenia across different populations, regions, and countries based on varying criteria. However, research using the latest KWGS diagnostic criteria, particularly concerning the prevalence of functional sarcopenia, is scarce.

Most previous studies have dichotomized fall history as either the presence or absence of any fall, overlooking the potential clinical differences between individuals who fall once and those who fall repeatedly. In this study, fall frequency was categorized into three tiers: single (≥1 fall/year), multiple (≥2 falls/year), and frequent (≥4 falls/year) falls. This classification aimed to reflect the increasing severity and clinical relevance of recurrent falls. Notably, the threshold of four or more falls was based on the SARC-F questionnaire, which identifies this cutoff as indicative of high sarcopenia risk. This categorization has also been supported by prior literature linking frequent falls with higher rates of functional decline, hospitalization, and institutionalization [13,14]. Therefore, stratifying fall frequency in this manner may enhance the clinical utility of sarcopenia screening and help identify individuals at greatest risk.

This cross-sectional study investigated the prevalence of sarcopenia and functional sarcopenia among community-dwelling older adults in Korea using the KWGS criteria. Additionally, we explored the association between sarcopenia, defined by various KWGS diagnostic components, and fall history, with separate analyses conducted according to sex. Therefore, this cross-sectional study aimed to (1) estimate the prevalence of sarcopenia, severe sarcopenia, and functional sarcopenia in community-dwelling older Korean adults using the newly established KWGS diagnostic criteria, and (2) evaluate the association between sarcopenia subtypes and fall risk across three thresholds (≥1, ≥2, and ≥4 falls), with analyses stratified by sex.

## 2. Materials and Methods

### 2.1. Study Population

**Figure 1:**
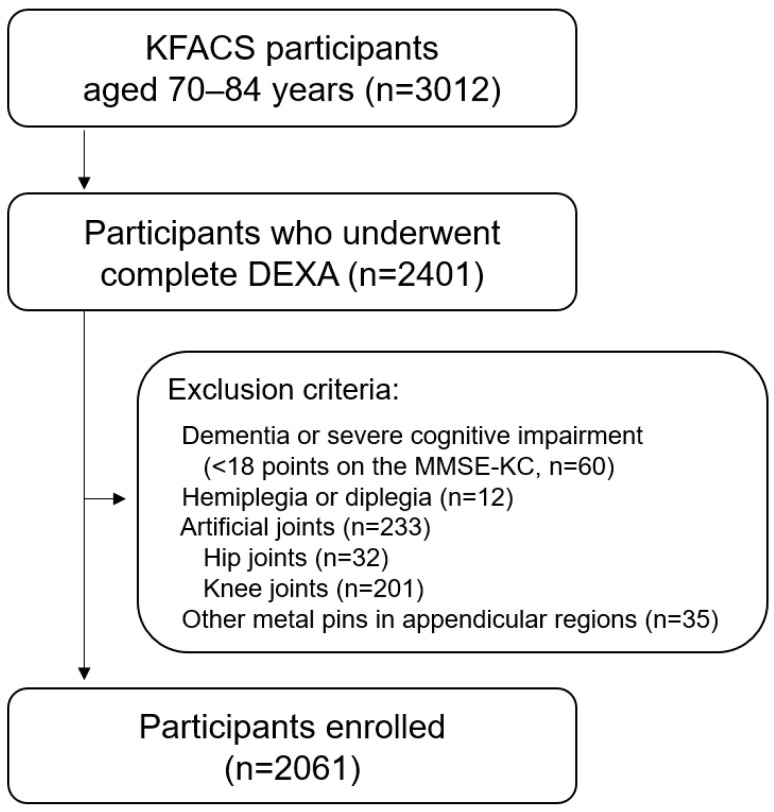
Flow chart of participant recruitment. KFACS, Korean Frailty and Aging Cohort Study; DEXA, dual-energy X-ray absorptiometry; MMSE-KC, Mini-Mental State Examination of the Korean version of the Consortium to Establish a Registry for Alzheimer’s Disease (CERAD) assessment packet.

Based on a cross-sectional design, this study analyzed data from the Korean Frailty and Aging Cohort Study (KFACS) conducted between 2016 and 2017. The KFACS is a nationwide initiative conducted across ten sites, including eight medical hospitals and two public health centers. The study initially enrolled 3012 older adults aged 70–84 years [15]. Participants at each site completed structured questionnaires, underwent face-to-face interviews, and underwent comprehensive health evaluations, including laboratory tests. Among the 3012 initially enrolled individuals, only those who underwent dual-energy X-ray absorptiometry (DEXA) for muscle mass measurement were eligible for sarcopenia assessment. Consequently, 2401 participants with complete DEXA data were included in the final analysis. The exclusion criteria were dementia or severe cognitive impairment (MMSE-KC score < 18) [16], hemiplegia or diplegia, and the presence of artificial joints or metallic implants in the appendicular skeleton (Figure 1). A formal sample size calculation was not performed, as this was a secondary analysis of pre-existing cohort data.

### 2.2. Measurement Methods and Criteria According to the KWGS Guidelines

As outlined in the KWGS guidelines, sarcopenia is characterized by a decrease in muscle mass accompanied by decreased muscle strength or reduced physical performance. Severe sarcopenia is identified when all three factors—diminished muscle mass, muscle strength, and physical performance—are present. Functional sarcopenia is defined as a condition in which muscle strength and physical performance are impaired, yet muscle mass remains within the normal range.

#### 2.2.1. Muscle Mass

Muscle mass was measured using DEXA, specifically focusing on appendicular lean muscle mass, excluding the bone. This value was adjusted for height by dividing by height squared, with a cutoff indicating low muscle mass set at <7.0 kg/m^2^ for men and <5.4 kg/m^2^ for women.

#### 2.2.2. Muscle Strength

Muscle strength was assessed by measuring the handgrip strength using a hand dynamometer (T.K.K.5401; Takei Scientific Instruments Co., Ltd., Tokyo, Japan). The participant stood with arms extended and was instructed to grip the device with maximum force for 3 s. Each arm was measured twice, and the highest recorded value was used for the evaluation. A grip strength of <28 kg for men and <18 kg for women was considered indicative of reduced muscle strength.

#### 2.2.3. Physical Performance

Physical performance was assessed using various tests, including the Short Physical Performance Battery (SPPB), gait speed, timed up-and-go (TUG) test, and chair stand test (CST). The SPPB consists of three components: gait speed, balance, and a five-time chair-stand test. Each component was scored from 0 to 4, with a total possible score of 12 points. A score of ≤9 was considered indicative of decreased physical performance. Gait speed was measured over 4 m at usual walking pace, with speeds below 1.0 m/s indicating decreased physical performance. The TUG test involves standing up from a chair, walking 3 m, returning, and sitting back down. Based on the lower 20% threshold for Koreans, times of ≥12 s were considered to reflect diminished physical performance. In the CST, participants were instructed to stand up from a seated position without using their arms and repeat this task five times as quickly as possible. A completion time of >10 s was considered indicative of reduced physical performance.

### 2.3. Fall Incidents in a Year

Fall incidents were assessed using self-reported data. Participants were asked whether they had fallen in the past year, and those who responded affirmatively were asked to report the total number of falls. Based on this information, fall frequency was categorized into three groups for analysis: ≥1 fall/year (one or more fall), ≥2 falls/year (multiple falls), and ≥4 falls/year (frequent falls). These thresholds were chosen based on established criteria in prior studies and clinical screening tools (e.g., SARC-F) [13,14].

### 2.4. Covariates

The following variables were included as covariates based on their potential to influence sarcopenia or fall risk: age, body mass index (BMI), hypertension, dyslipidemia, cerebrovascular disease, osteoarthritis, rheumatoid arthritis, osteoporosis, asthma, chronic obstructive pulmonary disease, diabetes mellitus, renal disease, and cancer [11,17].

### 2.5. Statistical Analysis

Descriptive statistics, frequency analysis, and chi-square tests were used. Continuous variables were compared using independent *t*-tests and are presented as mean ± standard deviation; other variables are presented as numbers and ratios (%). Logistic regression models were employed to examine associations between sarcopenia and fall risk, including both unadjusted and fully adjusted models. Odds ratios (ORs) and 95% confidence intervals (CIs) were calculated. The fully adjusted models controlled for the covariates listed in Section 2.4. All statistical analyses were conducted using the Statistical Package for Social Sciences (version 25.0; SPSS Inc., Chicago, IL, USA), and *p*-values < 0.05 were considered statistically significant. Forest plots were generated using Python (version 3.13.4) with the pandas library to visualize the fully adjusted ORs and 95% CIs for the association between sarcopenia definitions and fall frequency. Separate plots were created for men and women, and analyses were stratified by fall frequency thresholds (≥1, ≥2, and ≥4 falls per year).

## 3. Results

This study included 2061 participants, comprising 1040 men and 1021 women (Table 1). Among them, 391 individuals (158 men and 233 women) had experienced at least one fall in the previous year, whereas 132 participants (55 men and 77 women) reported falling two or more times within the same period; 41 participants (15 men and 26 women) reported experiencing four or more falls.

Table 2 shows the mean values and standard deviations of the components used in the diagnosis of sarcopenia in all participants. Handgrip strength was higher in men, whereas women exhibited a slower gait speed, poorer CST performance, and longer TUG test times. All the measured components showed statistically significant sex-based differences.

Table 3 presents the prevalence of sarcopenia, severe sarcopenia, and functional sarcopenia according to different diagnostic criteria. Among the physical performance measures derived from the KFACS data, the prevalence rates were calculated based on four assessment methods: SPPB, gait speed, TUG test, and CST. The prevalence of sarcopenia varies depending on the criteria used. The lowest prevalence was observed when sarcopenia was defined as reduced muscle mass combined with poor performance on the TUG test, affecting 8.5% of men and 5.6% of women. In contrast, the highest prevalence was found when the CST was used, with 26.7% of men and 18.7% of women meeting these criteria. When considering sarcopenia as meeting at least one of the physical performance criteria, the overall prevalence was 25.3%, with men (30.0%) exhibiting a higher prevalence than women (20.5%), across all assessment methods.

A similar pattern was observed in patients with severe sarcopenia. The lowest prevalence was recorded using the TUG test criteria, with 4.5% of men and 2.4% of women classified as having severe sarcopenia. In contrast, the CST criterion had the highest prevalence, affecting 10.1% of men and 5.0% of women. Across all the diagnostic criteria, severe sarcopenia was substantially more prevalent in men than in women, with an overall prevalence of 8.4%. Functional sarcopenia tended to be similar, with the lowest prevalence observed when using the TUG test and the highest when applying the CST. However, unlike sarcopenia and severe sarcopenia, functional sarcopenia was substantially more prevalent in women than in men. According to KWGS guidelines, the overall prevalence of functional sarcopenia is 7.8%.

**Figure 2:**
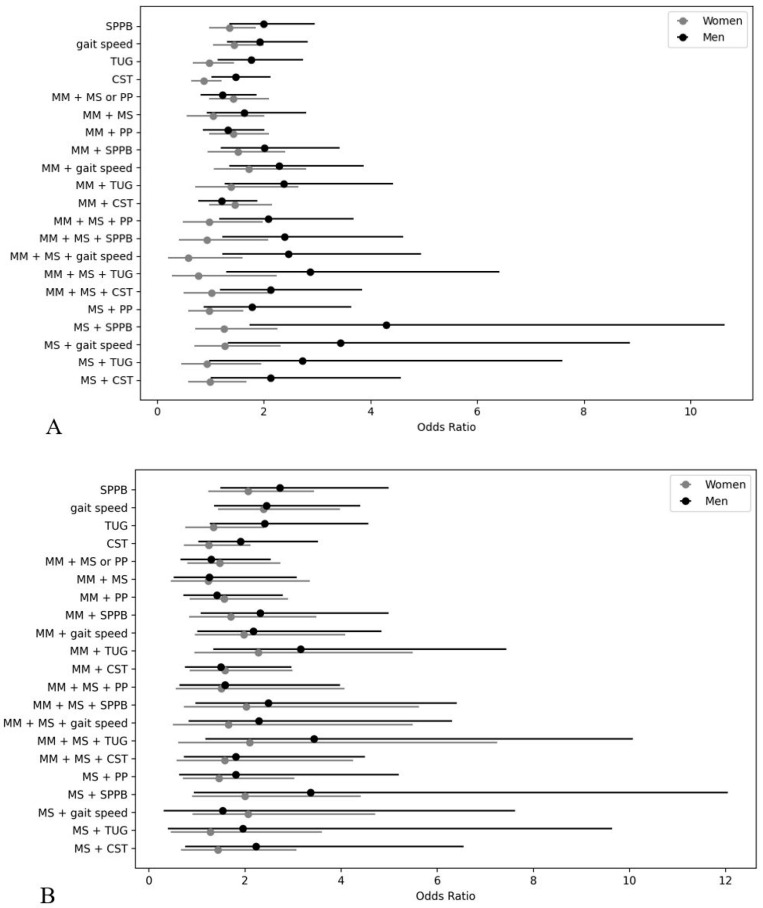

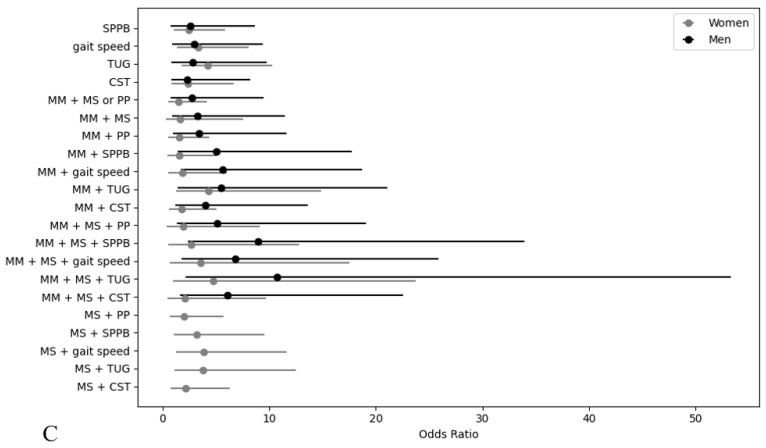
Forest plots of fully adjusted odds ratios and 95% confidence intervals for the association between sarcopenia, severe sarcopenia, and functional sarcopenia and fall experiences: (**A**) ≥1 fall/year, (**B**) ≥2 falls/year, and (**C**) ≥4 falls/year. MM: muscle mass; PP: physical performance; SPPB: Short Physical Performance Battery; TUG, timed up-and-go test; CST: chair stand test; MS: muscle strength.

Table 4, Table 5 and Table 6 and Figure 2 show the results of logistic regression analyses examining the association between sarcopenia, severe sarcopenia, and functional sarcopenia with fall incidence, using three fall thresholds: single, multiple, and frequent falls within the past year. In all three models, the results are presented separately for men and women and include both unadjusted and fully adjusted ORs with 95% CIs. Several definitions of sarcopenia were associated with fall risk, particularly in men (Table 4). Sarcopenia defined using low SPPB (OR = 2.01, CI: 1.20–3.42), slow gait speed (OR = 2.28, CI: 1.35–3.87), and prolonged TUG (OR = 2.37, CI: 1.27–4.43) showed elevated odds of falling. Severe sarcopenia combining low muscle mass, low muscle strength, and low physical performances was also associated with increased fall risk (OR = 2.08, CI: 1.17–3.68). Among the functional sarcopenia definitions, low SPPB and gait speed yielded the highest ORs in men (OR = 4.29, CI: 1.73–10.64 and OR = 3.43, CI: 1.32–8.86, respectively). In women, only sarcopenia, defined by slow gait speed, was associated with fall risk (OR = 1.72, CI: 1.06–2.80). No other adjusted models were statistically significant for women.

The association in men remained statistically significant for several diagnostic combinations (Table 5). Sarcopenia defined with low SPPB (OR = 2.32, CI: 1.08–4.99), slow gait speed (OR = 2.18, CI: 1.01–4.84), and prolonged TUG (OR = 3.16, CI: 1.34–7.44) were all associated with increased odds of falling. Severe sarcopenia defined using TUG (OR = 3.44, CI: 1.18–10.07) also showed a strong association. In women, gait speed-based sarcopenia definitions were weakly significant (OR = 1.98, CI: 0.96–4.09), but most other models did not reach statistical significance.

The association between sarcopenia and fall risk was stronger and more consistent in men (Table 6). Sarcopenia with slow gait speed (OR = 5.64, CI: 1.71–18.69), slow TUG (OR = 5.47, CI: 1.42–21.06), or low SPPB (OR = 5.07, CI: 1.45–17.79) showed increased odds of frequent falls. The definitions of severe sarcopenia consistently produced large effects in men. In women, functional sarcopenia, defined using low SPPB (OR = 3.20, CI: 1.07–9.57), slow gait speed (OR = 3.87, CI: 1.29–11.62), and prolonged TUG (OR = 3.79, CI: 1.15–12.55), was strongly associated with frequent falls.

## 4. Discussion

### 4.1. Main Findings

This study aimed to investigate the prevalence of sarcopenia, severe sarcopenia, and functional sarcopenia in the Korean population using the newly proposed 2023 KWGS diagnostic criteria. Unlike previous studies that utilized the AWGS 2019 guidelines, this study adopted the KWGS framework, which incorporates broader functional assessments, including the TUG test and revised CST thresholds. In addition to prevalence estimates, the present study also explored the association between sarcopenia and fall risk over the past year, considering three fall frequency thresholds (≥1, ≥2, and ≥4 falls) and stratifying the analyses by sex.

The prevalence of sarcopenia and severe sarcopenia according to the KWGS criteria was 32.9% and 10.1% in men and 21.5% and 5.0% in women, respectively. These estimates are slightly higher than those reported in previous studies using AWGS definitions with KFACS data, which reported a sarcopenia prevalence of 26.8% in men and 18.8% in women [18]. The higher prevalence observed in our study may be attributed to the inclusion of the TUG test as a physical performance criterion, and the use of stricter CST thresholds in the KWGS guidelines. Consistent with prior research, sarcopenia and severe sarcopenia were more prevalent in men than in women across all diagnostic combinations [19,20,21]. In contrast, the prevalence of functional sarcopenia, which is defined as low muscle strength and impaired physical performance despite normal muscle mass, was higher in women than in men, suggesting potential sex-based differences in muscle quality deterioration [22].

When examining the association between sarcopenia and fall risk, our findings demonstrate that the strength and statistical significance of these associations increased with fall frequency, particularly in men. In the single-fall group, sarcopenia definitions incorporating gait speed, TUG, and SPPB were strongly associated with fall risk, primarily in men. In the group with multiple falls, the pattern persisted, although the effect was slightly larger. The associations became most pronounced in the group with frequent falls, where multiple sarcopenia definitions, particularly those involving gait speed, were strongly associated with an increased fall risk in men. Severe sarcopenia, especially when combined with low gait speed or prolonged TUG test time, increases the odds of frequent falls.

Of the various sarcopenia diagnostic combinations, those incorporating gait speed consistently showed the strongest and most reliable association with fall risk across all fall thresholds. Compared to other functional measures, such as TUG or CST, gait speed not only demonstrated higher predictive values but also had narrower confidence intervals, reinforcing its clinical utility as a key indicator in fall risk screening. In women, although fewer associations reached statistical significance, functional sarcopenia definitions, particularly those defined using low gait speed and prolonged TUG, were strongly associated with frequent falls (≥4 falls/year). This suggests that while women may exhibit less muscle mass loss, functional impairments still play a crucial role in increasing fall risk, especially with recurrent falls.

This consistent association may be explained by the fact that gait speed reflects an integrated measure of neuromuscular coordination, balance, and lower limb strength [23,24,25,26,27]. Unlike the TUG test and CST, which assess more specific or isolated functions, gait speed captures the continuous and dynamic nature of mobility. It has also been linked to impaired sensorimotor control and increased gait variability, which contribute to the risk of falls.

The observed sex-based differences in both the prevalence and risk of falls may stem from underlying physiological and hormonal factors. Men tend to have greater absolute muscle mass and a higher proportion of fast-twitch type II fibers, which are more vulnerable to age-related atrophy [28,29,30]. This can lead to a substantial decline in strength and performance. In contrast, women often experience a more pronounced decline in muscle quality, with greater reductions in strength and function relative to muscle mass [31,32]. Hormonal differences, including reductions in testosterone and IGF-1 in men and estrogen in women, likely contribute to these sex-specific patterns [29,33,34,35,36]. Moreover, the application of uniform physical performance cutoffs across sexes, as is currently the case in most sarcopenia guidelines, including the KWGS, may not adequately reflect these biological differences [37,38,39]. In the current study, significant sex differences in mean physical performance measures were observed; however, the same thresholds for physical performance were applied for diagnostic classification in men and women [8]. These methodological limitations may have influenced both prevalence estimates and fall risk associations and warrant consideration in future revisions of the diagnostic criteria.

Another potential contributor is the method of adjusting the muscle mass. As the KWGS guidelines in this study adjusted appendicular muscle mass for height, overweight individuals may have been excluded despite potentially having impaired muscle function. Various studies indicate that sarcopenia prevalence varies widely depending on whether muscle mass is adjusted for height, weight, or BMI, with different methods yielding inconsistent results [40,41].

### 4.2. Clinical Implications

This study highlights meaningful sex differences in the prevalence of sarcopenia and its association with fall risk using the newly established KWGS diagnostic framework. Prevalence varied according to the functional test applied, with CST yielding the highest and TUG the lowest rates, respectively. In men, sarcopenia and severe sarcopenia were strongly associated with fall risk across all thresholds. In women, functional sarcopenia showed a stronger association with recurrent falls. These findings suggest that sex-specific and function-focused approaches may be beneficial in sarcopenia screening and prevention of falls.

### 4.3. Limitation, Strengths and Suggestions for Future Studies

This study had certain limitations. First, as this was a cross-sectional analysis, causality could not be established, highlighting the need for longitudinal studies to elucidate the relationship between sarcopenia and falls. Although the analyses were stratified by sex, further research is required to determine whether these findings can be generalized across different age groups and populations. In addition, some confounding variables may have been insufficiently or over-adjusted, potentially affecting the results. A notable limitation was that none of the male participants with functional sarcopenia had experienced four or more falls within the past year. The absence of events precluded the estimation of ORs for this subgroup, thus limiting the interpretation of sex-specific associations in high-frequency fallers with functional sarcopenia.

Moreover, potential behavioral and environmental factors that are known to influence fall risk—such as physical activity levels, polypharmacy, visual or vestibular impairments, medication side effects, and environmental hazards (e.g., slippery flooring or poor lighting)—were not fully captured in the dataset. The omission of these non-biological contributors may have introduced residual confounding, particularly in interpreting the independent effect of sarcopenia on fall outcomes. Future studies should aim to include these variables to enhance model accuracy and clinical interpretation.

Furthermore, the use of pre-collected data limited the inclusion of all relevant health conditions, and the exclusion of participants with artificial joints might have affected the evaluation of fall history. As this study utilized self-reported questionnaires to assess fall frequency over the past year, recall bias may have influenced the participants’ responses. Falls are accidental events that often lead to serious injuries, making them memorable. For this reason, self-reported data on multiple falls were considered reliable and used in the analysis.

Despite these limitations, this study has several strengths. It utilized a large, population-based cohort and applied the newly proposed national diagnostic criteria, which include updated physical performance metrics. The stratified analysis by fall frequency and sex also provided nuanced insights into clinically meaningful risk groups, which may inform targeted screening approaches. Future longitudinal studies are warranted to confirm the causal relationship between sarcopenia and falls, and to evaluate the contribution of individual diagnostic components to fall risk over time. Comparative research on different muscle mass adjustment methods and sex-specific performance thresholds is also needed to refine diagnostic criteria and improve clinical outcomes.

## 5. Conclusions

This study demonstrated that the prevalence and clinical relevance of sarcopenia, severe sarcopenia, and functional sarcopenia differed according to sex and diagnostic criteria when the 2023 KWGS guidelines were applied. Gait speed was the most consistent predictor of fall risk across all fall frequency thresholds, particularly in men, whereas functional sarcopenia was a stronger predictor of frequent falls in women. These findings support the importance of incorporating sex-specific and functional performance-based assessments in sarcopenia screening and underscore the need for longitudinal studies to clarify causality and optimize diagnostic strategies.

## Figures and Tables

**Table 1:** Baseline characteristics of the participants in a cross-sectional analysis of 2061 community-dwelling Korean adults aged 70–84 years.

Variables	Overall (*n* = 2061)	Men (*n* = 1040)	Women (*n* = 1021)
Age, years	75.87 ± 3.92	76.35 ± 3.93	75.36 ± 3.86
Height, cm	158.56 ± 8.43	164.97 ± 5.58	152.03 ± 5.21
Weight, kg	61.01 ± 9.32	65.21 ± 8.95	56.72 ± 7.58
BMI, kg/m^2^	24.22 ± 2.86	23.94 ± 2.86	24.52 ± 2.80
MMSE-KC	26.08 ± 2.71	26.51 ± 2.86	25.65 ± 2.88
Hypertension, *n* (%)	1145 (55.6%)	551 (53.0%)	594 (56.2%)
Dyslipidemia, *n* (%)	674 (32.7%)	251 (24.1%)	423 (41.4%)
Cardiovascular diseases, *n* (%)	92 (4.5%)	59 (5.7%)	33 (3.2%)
Osteoarthritis, *n* (%)	407 (19.7%)	111 (10.7%)	296 (29.0%)
Rheumatoid arthritis, *n* (%)	40 (1.9%)	9 (0.9%)	31 (3.0%)
Osteoporosis, *n* (%)	271 (13.1%)	29 (2.8%)	242 (23.7%)
Asthma, *n* (%)	65 (3.2%)	24 (2.3%)	41 (4.0%)
COPD, *n* (%)	26 (1.3%)	21 (2.0%)	5 (0.5%)
Diabetes mellitus, *n* (%)	459 (22.3%)	259 (24.9%)	200 (19.6%)
Kidney disease, *n* (%)	31 (1.5%)	21 (2.0%)	10 (1.0%)
Cancer, *n* (%)	72 (3.5%)	50 (4.8%)	22 (2.2%)
Number of prescription medications	3.36 ± 2.83	3.50 ± 3.00	3.21 ± 2.64
Number of over-the-counter medications	0.90 ± 1.23	0.71 ± 1.08	1.10 ± 1.33
Fall in a year, *n* (%)	391 (19.0%)	158 (15.2%)	233 (22.8%)
Two or more falls in a year, *n* (%)	132 (6.4%)	55 (5.3%)	77 (7.5%)
Three or more falls in a year, *n* (%)	70 (3.4%)	27 (2.6%)	43 (4.2%)
Four or more falls in a year, *n* (%)	41 (2.0%)	15 (1.4%)	26 (2.5%)
Fractures related to falls, *n* (%)	57 (2.8%)	25 (2.4%)	32 (3.1%)

Mean ± standard deviation; BMI: body mass index; MMSE-KC: Mini-Mental State Examination of the Korean version of the Consortium to Establish a Registry for Alzheimer’s Disease (CERAD) assessment packet; COPD: chronic obstructive pulmonary disease.

**Table 2:** Mean value of sarcopenia test components in a cross-sectional analysis of 2061 community-dwelling Korean adults aged 70–84 years.

Variables	Overall (*n* = 2061)	Men (*n* = 1040)	Women (*n* = 1021)	*p*-Value
SARC-F score	1.02 ± 1.43	0.62 ± 1.09	1.43 ± 1.61	<0.01 **
CC, cm	33.75 ± 2.83	34.62 ± 2.70	32.87 ± 2.69	<0.01 **
ASM, kg	16.35 ± 3.68	19.19 ± 2.70	13.47 ± 1.84	<0.01 **
Adjusted ASM, kg/m^2^	6.44 ± 0.99	7.04 ± 0.86	5.82 ± 0.71	<0.01 **
HGS, kg	26.88 ± 7.43	32.41 ± 5.74	21.25 ± 3.88	<0.01 **
Gait speed, m/s	1.13 ± 0.25	1.17 ± 0.26	1.09 ± 0.23	<0.01 **
CST, s	11.16 ± 3.63	10.50 ± 3.00	11.83 ± 4.07	<0.01 **
SPPB	10.07 ± 1.85	10.42 ± 1.66	9.72 ± 1.96	<0.01 **
TUG, s	10.22 ± 2.42	10.03 ± 2.24	10.41 ± 2.57	<0.01 **

Mean ± standard deviation; SARC-F score: strength, assistance with walking, rising from a chair, climbing stairs, and falls; CC: calf circumference; ASM: appendicular skeletal muscle mass; HGS: hand grip strength, CST: chair stand test; SPPB: Short Physical Performance Battery; TUG: timed up-and-go test. ** *p* < 0.01.

**Table 3:** Prevalence of sarcopenia, severe sarcopenia, and functional sarcopenia according to the KWGS guidelines.

Variables	Overall (*n* = 2061)	Men (*n* = 1040)	Women (*n* = 1021)	*p*-Value
Sarcopenia, *n* (%)				
MM + MS or PP †	562 (27.3%)	342 (32.9%)	220 (21.5%)	<0.01 **
MM + MS	215 (10.4%)	151 (14.5%)	64 (6.3%)	<0.01 **
MM + PP †	521 (25.3%)	312 (30.0%)	209 (20.5%)	<0.01 **
MM + SPPB	273 (13.2%)	149 (14.3%)	124 (12.1%)	0.14 *
MM + gait speed	242 (11.7%)	139 (13.3%)	104 (10.2%)	0.03 *
MM + TUG	145 (7.0%)	88 (8.5%)	57 (5.6%)	0.01 *
MM + CST	469 (22.8%)	278 (26.7%)	191 (18.7%)	<0.01 **
Severe sarcopenia, *n* (%)				
MM + MS + PP †	174 (8.4%)	121 (11.6%)	53 (5.2%)	<0.01 **
MM + MS + SPPB	118 (5.7%)	78 (7.5%)	40 (3.9%)	<0.01 **
MM + MS + gait speed	100 (4.9%)	68 (6.5%)	32 (3.1%)	0.01 *
MM + MS + TUG	72 (3.5%)	47 (4.5%)	25 (2.4%)	<0.01 **
MM + MS + CST	156 (7.6%)	105 (10.1%)	51 (5.0%)	<0.01 **
Functional sarcopenia, *n* (%)				
MS + PP †	160 (7.8%)	53 (5.1%)	107 (10.5%)	<0.01 **
MS + SPPB	98 (4.8%)	25 (2.4%)	73 (7.1%)	<0.01 **
MS + gait speed	89 (4.3%)	22 (2.1%)	67 (6.6%)	<0.01 **
MS + TUG	69 (3.3%)	20 (1.9%)	49 (4.8%)	<0.01 **
MS + CST	139 (6.7%)	43 (4.1%)	96 (9.4%)	<0.01 **

MM: muscle mass; PP: physical performance; SPPB: Short Physical Performance Battery; TUG: timed up-and-go test; CST: chair stand test; MS: muscle strength. † PP: at least one of the following criteria was met: SPPB, gait speed, TUG, or CST. * *p* < 0.05; ** *p* < 0.01

**Table 4:** Associations between sarcopenia, severe sarcopenia, and functional sarcopenia and single fall experiences (≥1 falls/year).

Variables	Unadjusted Model		Fully Adjusted Model ††	
	Men	Women	Men	Women
	OR (95% CI)	OR (95% CI)	OR (95% CI)	OR (95% CI)
Physical performance				
SPPB	2.13 (1.49–3.05) **	1.47 (1.10–1.98) *	2.00 (1.36–2.96) **	1.35 (0.98–1.85)
gait speed	2.11 (1.47–3.04) **	1.57 (1.16–2.12) **	1.92 (1.31–2.82) **	1.44 (1.05–1.99) *
TUG	1.92 (1.27–2.91) **	1.11 (0.78–1.60)	1.76 (1.14–2.73) *	0.98 (0.67–1.44)
CST	1.64 (1.16–2.32) *	0.96 (0.71–1.31)	1.47 (1.02–2.12) *	0.88 (0.64–1.21)
Sarcopenia				
MM + MS or PP †	1.29 (0.90–1.86)	1.38 (0.98–1.96)	1.23 (0.81–1.87)	1.43 (0.97–2.09)
MM + MS	1.70 (1.09–2.67) *	1.13 (0.62–2.07)	1.63 (0.94–2.80)	1.05 (0.55–2.01)
MM + PP †	1.42 (0.98–2.04)	1.38 (0.97–1.97)	1.32 (0.86–2.01)	1.43 (0.97–2.10)
MM + SPPB	1.94 (1.25–3.02) **	1.57 (1.03–2.40) *	2.01 (1.20–3.42) **	1.51 (0.95–2.40)
MM + gait speed	2.15 (1.40–3.36) **	1.79 (1.13–2.81) *	2.28 (1.35–3.87) **	1.72 (1.06–2.80) *
MM + TUG	2.24 (1.32–3.79) **	1.57 (0.86–2.85)	2.37 (1.27–4.43) **	1.38 (0.72–2.65)
MM + CST	1.32 (0.90–1.94)	1.42 (0.98–2.04)	1.21 (0.77–1.88)	1.45 (0.97–2.16)
Severe sarcopenia				
MM + MS + PP †	2.18 (1.37–3.47) **	1.08 (0.55–2.11)	2.08 (1.17–3.68) *	0.97 (0.48–1.98)
MM + MS + SPPB	2.33 (1.35–4.04) **	1.07 (0.50–2.30)	2.39 (1.23–4.62) *	0.93 (0.41–2.08)
MM + MS + gait speed	2.45 (1.38–4.36) **	0.68 (0.26–1.81)	2.46 (1.22–4.95) *	0.58 (0.21–1.60)
MM + MS + TUG	2.69 (1.38–5.22) **	0.92 (0.34–2.50)	2.87 (1.29–6.42) *	0.78 (0.28–2.24)
MM + MS + CST	2.30 (1.41–3.75) **	1.14 (0.58–2.22)	2.13 (1.18–3.85) *	1.02 (0.50–2.09)
Functional sarcopenia				
MS + PP †	1.85 (0.93–3.66)	1.13 (0.69–1.82)	1.78 (0.87–3.64)	0.97 (0.58–1.62)
MS + SPPB	4.22 (1.84–9.69) **	1.49 (0.87–2.55)	4.29 (1.73–10.64) **	1.26 (0.71–2.26)
MS + gait speed	3.62 (1.48–8.87) **	1.46 (0.83–2.56)	3.43 (1.32–8.86) *	1.27 (0.70–2.32)
MS + TUG	2.71 (1.01–7.25) *	1.07 (0.53–2.14)	2.72 (0.98–7.59)	0.94 (0.45–1.95)
MS + CST	2.18 (1.06–4.47) *	1.16 (0.70–1.92)	2.13 (1.00–4.57)	0.99 (0.58–1.68)

OR, odds ratio; CI: confidence interval; MM: muscle mass; PP: physical performance; SPPB: Short Physical Performance Battery; TUG, timed up-and-go test; CST: chair stand test; MS: muscle strength. † PP: at least one of the following criteria was met: SPPB, gait speed, TUG, or CST. †† Fully adjusted model adjusted for age, body mass index (BMI), hypertension, dyslipidemia, cerebrovascular disease, osteoarthritis, rheumatoid arthritis, osteoporosis, asthma, chronic obstructive pulmonary disease, diabetes mellitus, renal disease, and cancer. * *p* < 0.05; ** *p* < 0.01.

**Table 5:** Associations between sarcopenia, severe sarcopenia, and functional sarcopenia and multiple fall experiences (≥2 falls/year).

Variables	Unadjusted Model		Fully Adjusted Model ††	
	Men	Women	Men	Women
	OR (95% CI)	OR (95% CI)	OR (95% CI)	OR (95% CI)
Physical performance				
SPPB	2.93 (1.69–5.07) **	2.25 (1.41–3.61) **	2.73 (1.49–4.99) **	2.07 (1.24–3.44) **
gait speed	2.71 (1.56–4.71) **	2.51 (1.57–4.02) **	2.45 (1.36–4.40) **	2.39 (1.44–3.98) **
TUG	2.73 (1.50–4.98) **	1.48 (0.87–2.52)	2.41 (1.27–4.57) **	1.35 (0.76–2.40)
CST	2.15 (1.20–3.84) *	1.38 (0.83–2.28)	1.91(1.03–3.52) *	1.25 (0.73–2.12)
Sarcopenia				
MM + MS or PP †	1.39 (0.78–2.48)	1.44 (0.83–2.50)	1.30 (0.66–2.54)	1.48 (0.80–2.74)
MM + MS	1.51 (0.72–3.16)	1.49 (0.61–3.65)	1.26 (0.52–3.08)	1.24 (0.46–3.35)
MM + PP †	1.53 (0.86–2.73)	1.53 (0.88–2.65)	1.42 (0.72–2.79)	1.57 (0.85–2.90)
MM + SPPB	2.74 (1.46–5.12) **	1.84 (0.98–3.46)	2.32 (1.08–4.99) *	1.71 (0.84–3.49)
MM + gait speed	2.40 (1.23–4.67) *	2.06 (1.07–3.97) *	2.18 (1.01–4.84) *	1.98 (0.96–4.09)
MM + TUG	3.68 (1.83–7.39) **	2.70 (1.25–5.86) *	3.16 (1.34–7.44) **	2.28 (0.95–5.49)
MM + CST	1.65 (0.92–2.96)	1.59 (0.91–2.80)	1.50 (0.75–2.97)	1.59 (0.85–2.99)
Severe sarcopenia				
MM + MS + PP †	1.91 (0.91–4.04)	1.84 (0.75–4.54)	1.59 (0.64–3.98)	1.51 (0.56–4.07)
MM + MS + SPPB	3.12 (1.46–6.9) **	2.55 (1.02–6.38) *	2.49 (0.97–6.41)	2.03 (0.73–5.62)
MM + MS + gait speed	2.83 (1.24–6.47) *	2.06 (0.69–6.13)	2.29 (0.83–6.31)	1.66 (0.50–5.49)
MM + MS + TUG	4.36 (1.87–10.16) **	2.75 (0.90–8.35)	3.44 (1.18–10.07) *	2.10 (0.61–7.25)
MM + MS + CST	2.24 (1.06–4.74) *	1.92 (0.78–4.75)	1.81 (0.73–4.50)	1.58 (0.58–4.28)
Functional sarcopenia				
MS + PP †	2.21 (0.82–5.98)	1.82 (0.93–3.59)	1.81 (0.63–5.20)	1.46 (0.71–3.03)
MS + SPPB	4.05 (1.30–12.55) *	2.56 (1.26–5.19) **	3.37 (0.94–12.05)	2.00 (0.90–4.41)
MS + gait speed	2.12 (0.47–9.52)	2.53 (1.21–5.29) *	1.54 (0.31–7.62)	2.07 (0.91–4.71)
MS + TUG	2.36 (0.52–10.66)	1.64 (0.62–4.34)	1.96 (0.40–9.64)	1.28 (0.46–3.60)
MS + CST	2.80 (1.02–7.63) *	1.87 (0.93–3.75)	2.23 (0.76–6.55)	1.44 (0.67–3.07)

OR, odds ratio; CI: confidence interval; MM: muscle mass; PP: physical performance; SPPB: Short Physical Performance Battery; TUG, timed up-and-go test; CST: chair stand test; MS: muscle strength. † PP: at least one of the following criteria was met: SPPB, gait speed, TUG, or CST. †† Fully adjusted model adjusted for age, body mass index (BMI), hypertension, dyslipidemia, cerebrovascular disease, osteoarthritis, rheumatoid arthritis, osteoporosis, asthma, chronic obstructive pulmonary disease, diabetes mellitus, renal disease, and cancer. * *p* < 0.05; ** *p* < 0.01.

**Table 6:** Associations between sarcopenia, severe sarcopenia, and functional sarcopenia and frequent fall experiences (≥4 falls/year).

Variables	Unadjusted Model		Fully Adjusted Model ††	
	Men	Women	Men	Women
	OR (95% CI)	OR (95% CI)	OR (95% CI)	OR (95% CI)
Physical performance				
SPPB	3.56 (1.28–9.92) *	2.60 (1.17–5.79) *	2.61 (0.78–8.66)	2.46 (1.03–5.89) *
gait speed	3.84 (1.38–10.71) *	3.21 (1.44–7.15) **	2.99 (0.95–9.40)	3.35 (1.38–8.14) **
TUG	3.80 (1.33–10.82) *	3.66 (1.66–8.03) **	2.87 (0.84–9.76)	4.27 (1.77–10.33) **
CST	2.88 (0.91–9.11)	2.45 (0.92–6.55)	2.35 (0.87–8.21)	2.37 (0.84–6.65)
Sarcopenia				
MM + MS or PP †	4.18 (1.42–12.31) *	1.10 (0.43–2.76)	2.73 (0.78–9.51)	1.51 (0.54–4.17)
MM + MS	5.35 (1.91–14.99) **	1.25 (0.29–5.43)	3.28 (0.94–11.47)	1.63 (0.35–7.59)
MM + PP †	4.79 (1.62–14.13) **	1.17 (0.46–2.95)	3.42 (0.99–11.60)	1.59 (0.58–4.42)
MM + SPPB	7.17 (2.56–20.07) **	1.33 (0.45–3.91)	5.07 (1.45–17.79) *	1.56 (0.48–5.03)
MM + gait speed	7.87 (2.81–22.06) **	1.63 (0.55–4.82)	5.64 (1.71–18.69) **	1.87 (0.58–6.03)
MM + TUG	7.67 (2.66–22.07) **	3.23 (1.08–9.72) *	5.47 (1.42–21.06) *	4.33 (1.26–14.86) *
MM + CST	5.65 (1.91–16.68) **	1.31 (0.52–3.32)	4.04 (1.20–13.65) *	1.81 (0.65–5.04)
Severe sarcopenia				
MM + MS + PP †	6.99 (2.49–19.64) **	1.54 (0.36–6.71)	5.14 (1.38–19.09) *	1.95 (0.42–9.10)
MM + MS + SPPB	11.76 (4.15–33.35) **	2.10 (0.48–9.21)	8.95 (2.36–33.92) **	2.70 (0.57–12.84)
MM + MS + gait speed	10.36 (3.57–30.02) **	2.68 (0.61–11.87)	6.86 (1.82–25.86) **	3.57 (0.73–17.53)
MM + MS + TUG	11.70 (3.83–35.76) **	3.52 (0.79–15.79)	10.76 (2.17–53.31) **	4.79 (0.96–23.76)
MM + MS + CST	8.28 (2.94–23.31) **	1.61 (0.37–7.00)	6.11 (1.66–22.53) **	2.08 (0.45–9.73)
Functional sarcopenia				
MS + PP †	—	2.09 (0.77–5.65)	—	2.00 (0.70–5.71)
MS + SPPB	—	3.25 (1.20–8.88) *	—	3.20 (1.07–9.57) *
MS + gait speed	—	3.58 (1.31–9.82) *	—	3.87 (1.29–11.62) *
MS + TUG	—	3.84 (1.27–11.61) *	—	3.79 (1.15–12.55) *
MS + CST	—	2.37 (0.87–5.42)	—	2.21 (0.77–6.34)

OR, odds ratio; CI: confidence interval; MM: muscle mass; PP: physical performance; SPPB: Short Physical Performance Battery; TUG, timed up-and-go test; CST: chair stand test; MS: muscle strength. —: Odds ratios could not be calculated because none of the male participants with functional sarcopenia experienced four or more falls. † PP: at least one of the following criteria was met: SPPB, gait speed, TUG, or CST. †† Fully adjusted model adjusted for age, body mass index (BMI), hypertension, dyslipidemia, cerebrovascular disease, osteoarthritis, rheumatoid arthritis, osteoporosis, asthma, chronic obstructive pulmonary disease, diabetes mellitus, renal disease, and cancer. * *p* < 0.05; ** *p* < 0.01.

## Data Availability

All cohort data supporting the findings of this study are available from the KFACS to all researchers upon reasonable request. All news articles published in the KFACS database, data provision manuals, and contact information are available on the KFACS website (http://www.kfacs.kr).

## Abbreviations

EWGSOP, European Working Group on Sarcopenia in Older People; AWGS, Asian Working Group for Sarcopenia; KWGS, Korean Working Group on Sarcopenia; KFACS, Korean Frailty and Aging Cohort Study; DEXA, dual-energy X-ray absorptiometry; OR, odds ratio; CI, confidence interval; SPPB, Short Physical Performance Battery; TUG, timed up-and-go test; and CST, chair stand test.

## References

[1] Cruz-Jentoft A.J., Sayer A.A. (2019). Sarcopenia. Lancet.

[2] Santilli V., Bernetti A., Mangone M., Paoloni M. (2014). Clinical definition of sarcopenia. Clin. Cases Miner. Bone Metab..

[3] Muscaritoli M., Anker S.D., Argilés J., Aversa Z., Bauer J., Biolo G., Boirie Y., Bosaeus I., Cederholm T., Costelli P. (2010). Consensus definition of sarcopenia, cachexia and pre-cachexia: Joint document elaborated by Special Interest Groups (SIG)“cachexia-anorexia in chronic wasting diseases” and “nutrition in geriatrics”. Clin. Nutr..

[4] Morley J. (2008). Sarcopenia: Diagnosis and treatment. J. Nutr. Health Aging.

[5] Wallengren O., Bosaeus I., Frändin K., Lissner L., Falk Erhag H., Wetterberg H., Sterner T.R., Rydén L., Rothenberg E., Skoog I. (2021). Comparison of the 2010 and 2019 diagnostic criteria for sarcopenia by the European Working Group on Sarcopenia in Older People (EWGSOP) in two cohorts of Swedish older adults. BMC Geriatr..

[6] Cruz-Jentoft A.J., Baeyens J.P., Bauer J.M., Boirie Y., Cederholm T., Landi F., Martin F.C., Michel J.-P., Rolland Y., Schneider S.M. (2010). Sarcopenia: European consensus on definition and diagnosis: Report of the European Working Group on Sarcopenia in Older People. Age Ageing.

[7] Chen L.-K., Woo J., Assantachai P., Auyeung T.-W., Chou M.-Y., Iijima K., Jang H.C., Kang L., Kim M., Kim S. (2020). Asian Working Group for Sarcopenia: 2019 consensus update on sarcopenia diagnosis and treatment. J. Am. Med. Dir. Assoc..

[8] Baek J.Y., Jung H.-W., Kim K.M., Kim M., Park C.Y., Lee K.-P., Lee S.Y., Jang I.-Y., Jeon O.H., Lim J.-Y. (2023). Korean Working Group on Sarcopenia guideline: Expert consensus on sarcopenia screening and diagnosis by the Korean Society of Sarcopenia, the Korean Society for Bone and Mineral Research, and the Korean Geriatrics Society. Ann. Geriatr. Med. Res..

[9] Dionyssiotis Y. (2012). Analyzing the problem of falls among older people. Int. J. Gen. Med..

[10] Ganz D.A., Bao Y., Shekelle P.G., Rubenstein L.Z. (2007). Will my patient fall?. JAMA.

[11] Yeung S.S., Reijnierse E.M., Pham V.K., Trappenburg M.C., Lim W.K., Meskers C.G., Maier A.B. (2019). Sarcopenia and its association with falls and fractures in older adults: A systematic review and meta-analysis. J. Cachexia Sarcopenia Muscle.

[12] Zhang Y., Hao Q., Ge M., Dong B. (2018). Association of sarcopenia and fractures in community-dwelling older adults: A systematic review and meta-analysis of cohort studies. Osteoporos. Int..

[13] Malmstrom T.K., Miller D.K., Simonsick E.M., Ferrucci L., Morley J.E. (2016). SARC-F: A symptom score to predict persons with sarcopenia at risk for poor functional outcomes. J. Cachexia Sarcopenia Muscle.

[14] Malmstrom T.K., Morley J.E. (2013). SARC-F: A simple questionnaire to rapidly diagnose sarcopenia. J. Am. Med. Dir. Assoc..

[15] Won C.W., Lee S., Kim J., Chon D., Kim S., Kim C.-O., Kim M.K., Cho B., Choi K.M., Roh E. (2020). Korean frailty and aging cohort study (KFACS): Cohort profile. BMJ Open.

[16] Pezzotti P., Scalmana S., Mastromattei A., Di Lallo D., Group P.A.W. (2008). The accuracy of the MMSE in detecting cognitive impairment when administered by general practitioners: A prospective observational study. BMC Fam. Pract..

[17] Gao Q., Hu K., Yan C., Zhao B., Mei F., Chen F., Zhao L., Shang Y., Ma Y., Ma B. (2021). Associated factors of sarcopenia in community-dwelling older adults: A systematic review and meta-analysis. Nutrients.

[18] Kim M., Won C.W. (2020). Sarcopenia in Korean community-dwelling adults aged 70 years and older: Application of screening and diagnostic tools from the Asian Working Group for Sarcopenia 2019 update. J. Am. Med. Dir. Assoc..

[19] Choo Y.J., Chang M.C. (2021). Prevalence of sarcopenia among the elderly in Korea: A meta-analysis. J. Prev. Med. Public Health.

[20] Kim S., Ha Y.-C., Kim D.-Y., Yoo J.-I. (2024). Recent Update on the Prevalence of Sarcopenia in Koreans: Findings from the Korea National Health and Nutrition Examination Survey. J. Bone Metab..

[21] Spira D., Norman K., Nikolov J., Demuth I., Steinhagen-Thiessen E., Eckardt R. (2016). Prevalence and definition of sarcopenia in community dwelling older people. Z. Für Gerontol. Und Geriatr..

[22] Doherty T.J. (2001). The influence of aging and sex on skeletal muscle mass and strength. Curr. Opin. Clin. Nutr. Metab. Care.

[23] Pettersson B., Nordin E., Ramnemark A., Lundin-Olsson L. (2020). Neither Timed Up and Go test nor Short Physical Performance Battery predict future falls among independent adults aged≥ 75 years living in the community. J. Frailty Sarcopenia Falls.

[24] Kyrdalen I.L., Thingstad P., Sandvik L., Ormstad H. (2019). Associations between gait speed and well-known fall risk factors among community-dwelling older adults. Physiother. Res. Int..

[25] Freiberger E., Fabbietti P., Corsonello A., Lattanzio F., Sieber C., Tap L., Mattace-Raso F., Ärnlöv J., Carlsson A.C., Roller-Wirnsberger R. (2024). Short physical performance battery is not associated with falls and injurious falls in older persons: Longitudinal data of the SCOPE project. Eur. Geriatr. Med..

[26] Studenski S., Perera S., Patel K., Rosano C., Faulkner K., Inzitari M., Brach J., Chandler J., Cawthon P., Connor E.B. (2011). Gait speed and survival in older adults. JAMA.

[27] Verghese J., Holtzer R., Lipton R.B., Wang C. (2009). Quantitative gait markers and incident fall risk in older adults. J. Gerontol. Ser. A Biomed. Sci. Med. Sci..

[28] Narici M.V., Maffulli N. (2010). Sarcopenia: Characteristics, mechanisms and functional significance. Br. Med. Bull..

[29] Tay L., Ding Y., Leung B., Ismail N., Yeo A., Yew S., Tay K., Tan C., Chong M. (2015). Sex-specific differences in risk factors for sarcopenia amongst community-dwelling older adults. Age.

[30] Liu C., Cheng K.Y.-K., Tong X., Cheung W.-H., Chow S.K.-H., Law S.W., Wong R.M.Y. (2023). The role of obesity in sarcopenia and the optimal body composition to prevent against sarcopenia and obesity. Front. Endocrinol..

[31] Wu Y.-H., Hwang A.-C., Liu L.-K., Peng L.-N., Chen L.-K. (2016). Sex differences of sarcopenia in Asian populations: The implications in diagnosis and management. J. Clin. Gerontol. Geriatr..

[32] Schaap L.A., Van Schoor N.M., Lips P., Visser M. (2018). Associations of sarcopenia definitions, and their components, with the incidence of recurrent falling and fractures: The longitudinal aging study Amsterdam. J. Gerontol. Ser. A.

[33] Bian A., Ma Y., Zhou X., Guo Y., Wang W., Zhang Y., Wang X. (2020). Association between sarcopenia and levels of growth hormone and insulin-like growth factor-1 in the elderly. BMC Musculoskelet. Disord..

[34] Kirchengast S., Huber J. (2009). Gender and age differences in lean soft tissue mass and sarcopenia among healthy elderly. Anthropol. Anz..

[35] de Jong J.C., Attema B.J., van der Hoek M.D., Verschuren L., Caspers M.P., Kleemann R., van der Leij F.R., van den Hoek A.M., Nieuwenhuizen A.G., Keijer J. (2023). Sex differences in skeletal muscle-aging trajectory: Same processes, but with a different ranking. Geroscience.

[36] Haizlip K., Harrison B., Leinwand L. (2015). Sex-based differences in skeletal muscle kinetics and fiber-type composition. Physiology.

[37] Pagotto V., Silveira E.A. (2014). Methods, diagnostic criteria, cutoff points, and prevalence of sarcopenia among older people. Sci. World J..

[38] Park C.-H., Do J.G., Lee Y.-T., Yoon K.J. (2022). Sex difference in cutoff and prevalence of sarcopenia among 300,090 Urban Korean population: Association with metabolic syndrome. Medicina.

[39] Sialino L.D., Schaap L.A., van Oostrom S.H., Picavet H.S.J., Twisk J.W., Verschuren W.M., Visser M., Wijnhoven H.A. (2021). The sex difference in gait speed among older adults: How do sociodemographic, lifestyle, social and health determinants contribute?. BMC Geriatr..

[40] Kinoshita K., Satake S., Matsui Y., Arai H. (2021). Association between sarcopenia and fall risk according to the muscle mass adjustment method in Japanese older outpatients. J. Nutr. Health Aging.

[41] Studenski S.A., Peters K.W., Alley D.E., Cawthon P.M., McLean R.R., Harris T.B., Ferrucci L., Guralnik J.M., Fragala M.S., Kenny A.M. (2014). The FNIH sarcopenia project: Rationale, study description, conference recommendations, and final estimates. J. Gerontol. Ser. A Biomed. Sci. Med. Sci..

